# Management of idiopathic granulomatous mastitis: dilemmas in diagnosis and treatment

**DOI:** 10.1186/1471-2482-14-66

**Published:** 2014-09-04

**Authors:** Gulten Kiyak, Ersin Gurkan Dumlu, Ibrahim Kilinc, Mehmet Tokaç, Soner Akbaba, Ahmet Gurer, Alper Bilal Ozkardes, Mehmet Kilic

**Affiliations:** 1 General Surgery Department, Atatürk Research and Training Hospital, 06800 Ankara, Turkey; 2 Department of General Surgery, Yildirim Beyazit Research and Training Hospital, Ankara, Turkey; 3 Faculty of Medicine Department of General Surgery, Yildirim Beyazit University, Ankara, Turkey

**Keywords:** Beast, Granulomatous, Mastitis, Diagnosis, Treatment

## Abstract

**Background:**

Idiopathic granulomatous mastitis (IGM) is a rare inflammatory disease.

Because of it’s uncommon etiology and rareness, diagnosis and treatment is still a challenge. Owing to wide spectrum of IGM it is difficult to standardize and optimize the treatment. The aim of this study was to report and describe the clinical signs, radiological findings, management, clinical course and the recurrence rate of the patients which were treated due to IGM.

**Methods:**

In this retrospective review of patients diagnosed with IGM histopathologically between January 2006 and December 2011, medical reports, ultrasonography (US) and mammograhy (MMG) findings, follow-up information and recurrence were obtained from records.

**Results:**

Painful, firm and ill defined mass was the symptom of all patients. While parenchymal heterogeneity, abscess and mass were the findings of US, increased asymmetric density was the main finding of MMG. Wide local excision was performed in 15 (62.5%) patients, incisional biopsy with abscess drainage was performed in 9 (37.5%) patients. Median follow-up was 34.8 (range 10–66) months.

**Conclusions:**

While the physical examination give rise to thought of breast carcinoma, the appearance of parenchymal heterogeneity and abscess formation on US especially with enlarged axillary lymph nodes support the presence of an inflammatory process. But these findings do not exclude carcinoma. Hereby, histopathologic confirmation is mandatory to ensure that a malignancy is not missed.

## Background

IGM was first described by Kessler and Wolloch in 1972 [1]. IGM is a rare inflammatory disease of the breast, but it is important because of two reasons. First clinical and radiological findings of IGM mimicks breast carcinoma and the differential diagnosis can only be confirmed histopathologically. Second, it is difficult to treat IGM especially if presented with fistula and abscess formation. Because of it’s unclear etiology and rareness, diagnosis and treatment is still a challenge [2]. An optimal treatment has not been yet established, while medical therapy, wide local excision and abscess drainage are currently the favored treatment options for IGM [3]. The aim of this study was to report and describe the clinical signs, radiological findings, management, clinical course and the recurrence ratio of the patients which were treated due to IGM.

## Methods

We reviewed records of patients diagnosed with IGM histopathologically between January 2006 and December 2011 in this retrospective study. The design of the study has been approved by Yildirim Beyazit University Faculty of Medicine ethics committee thus meets the standards of the Declaration of Helsinki. Due to retrospective nature of the study, the ethics committee did not require a written consent from the participants.

Medical reports about the patient’s complaints, presence of pregnancy, number of pregnancies, use of contraceptives, follow-up informations and recurrence were obtained from clinical records. All of the patients underwent a clinical breast examination. An US was performed on all patients. A MMG was obtained from patients older than 40 years. A magnetic resonance imaging (MRI) was performed in one patient.

Histopathological diagnosis was obtained from incisional or wide local excisional biopsies. Wide local excision was performed for a minimum lesion-free radial margin 5–10 mm. Incisional biopsy was obtained from patients with abscess formation. After drainage of abscess, incisional biopsy was performed from abscess cavity and obtained at least four tissue samples. If there was fistula formation to the skin, affected skin was excised too.

Inflammatory reaction with granulomas which composed of epitheloid histiocytes, Langhans giant cells accompanied by lymphocytes, plasma cells and occasional eosinophils centered on lobules was diagnosed with IGM on histopathologic examination. All aspirates and tissue samples were examined with hematoxylin-eosin staining procedure as well as special stains for tuberculosis and fungal infection. Cultures for aerob bacteries (streptococcus, staphylococcus, enterococcus, klebsiella, pseudomonas, e. coli..), anaerob bacteries (clostridium, bacteroides..), were performed for the patients with abscess formation. US and physical examination were performed every month until resolution of lesions was confirmed.

## Results

24 patients diagnosed with IGM histopathologically and had complete follow-up data has been evaluated and included in the study. The mean age was 38.4 years ranged from 28 to 60 years. 87.5% of patients (n = 21) were at reproductive ages. All patients had children. None of the patients had a history of oral contraseptive use and lactacion in the last one year. Painful , firm and ill defined mass was the symptom of all patients. Painful mass lesions were accompanied with skin changes such as erythema and edema in 16 (66.6%) patients. The left breast was affected in 13 (54.1%) patients, right breast in 9 (37.5%) patients and bilateral in 2 (8.3%) patients. The lesions were located in upper external quadrant in 5 (20.8%) patients, in upper internal quadrant in 1 patient (4.2%) in inferior internal quadrant in 1 patient (4,2%), in inferior external quadrant in 3 (12.5%) patients, in periareolar region in 7 patients (29.2%) and diffuse involvement was seen in 7 patients (29.2%) (Table 1).

**Table 1:** Location of lesions in the breast

**Location of lesions in the breast**	**No. (%)**
Periareolar region	7 (29.2)
Diffuse	7 (29.2)
Upper external quadran	5 (20.8)
Inferior external quadran	3 (12.5)
Upper internal quadran	1 (4.2)
Inferior internal quadran	1 (4.2)

**Figure 1:**
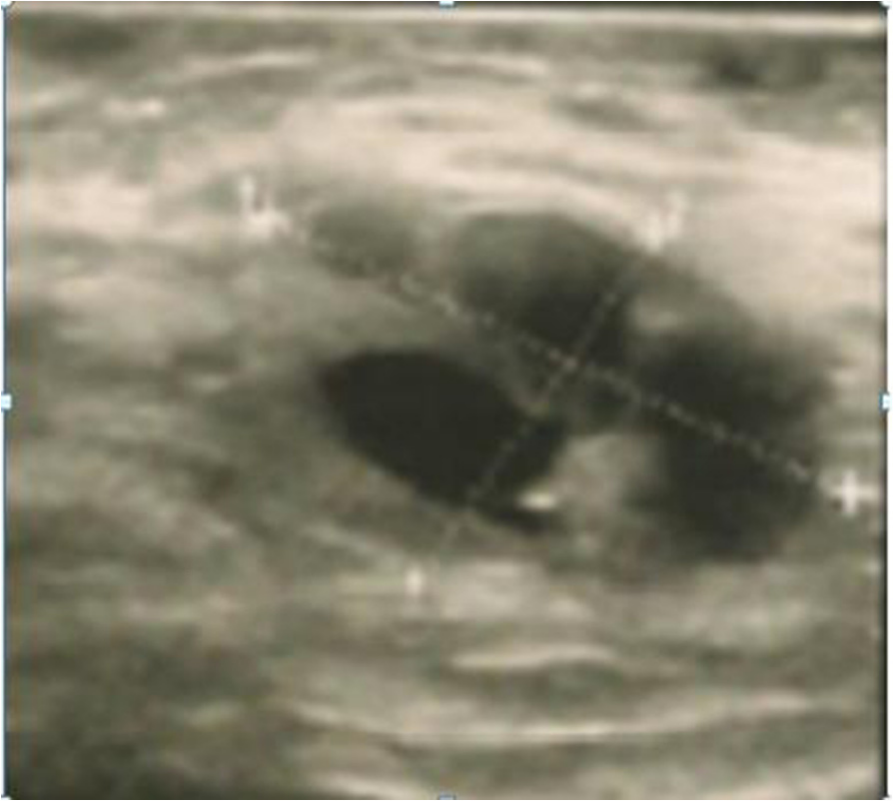
Transvers US scan shows cystic lesion with thickened wall consistent with abscess formation.

Parenchymal heterogeneity with no discrete mass had been established in 7 patients (29.1%), irregular mass in 7 patients (29.1%), abscess formation in 4 patients (16.6%), irregular mass with heterogeneity in 1 patient (4.2%), heterogeneity with abscess formation in 4 patients (16.6%), heterogeneity, abscess formation and mass in 1 patient (4.2%) on US (Table 2) (Figure 1). Associated enlarged axillary lymph nodes were present in 14 (41.7%) patients.

**Table 2:** US findings of patients with IGM

**US findings**	**No. (%)**
Parenchymal heterogeneity	7 (29.1)
Irregular mass	7 (29.1)
Abscess formation	4 (16.6)
Irregular mass + heterogeneity	1 (4.2)
Abscess formation + heterogeneity	4 (16.6)
Abscess formation + heterogeneity + mass	1 (4.2)

**Figure 2:**
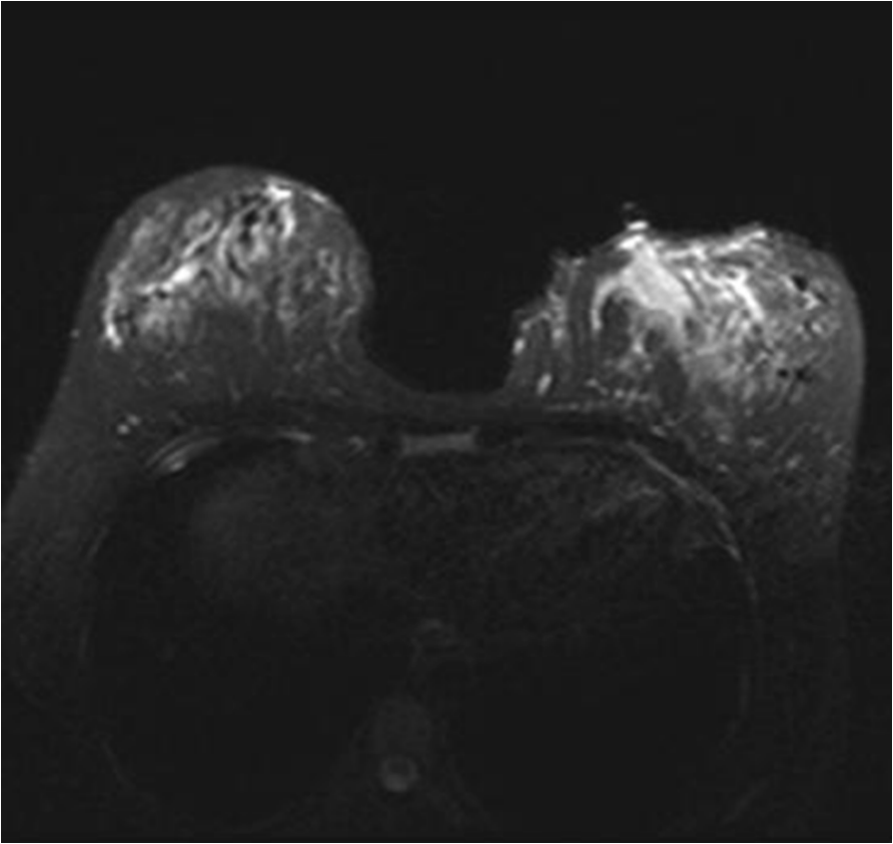
**T2 weighted fat saturated image demonstrate an irregular non-mass lesion in lower iner quadrant in left breast showing high signal intensity.** After administration of Gadalinium based contrast agent (L kont T1) the lesion enchanced partly in type I and in type II pattern heterogenously.

MMG was obtained from 7 patients. MMG detected increased asymmetric density in 3 patients, asymmetric density with skin thickening in 2 patients and asymmetric density with skin thickening and nipple inversion in 2 patients.MRI was performed in one patient which involvement was diffuse and bilateral and resistant to the treatment (Figure 2). MRI was needed for this patient to obtain additional information.

Wide local excision was performed in 15 (62.5%) patients, incisional biopsy with abscess drainage was performed in 9 (37.5%) patients. Each case demonstrated the presence of epithelioid histiocytes, lymphocytes, plasma cells, polymorphonuclear leukocytes and multinucleated Langhans-type giant cells without caseous necrosis. Serologic and bacterial tests were negative in all patients. But, patients with abscess formation received amoxcisilin 875 mg and clavulanic acid 125 mg.

Median follow-up was 34.8 (range 10–66) months. Treatment with wide local excision could be successfully performed in 15 patients. There was recurrence after 24 months in one patient in another quadrant of the breast and treated with reexcision. The mean interval of resolution in patients with abscess formation was 5.1 months (range 3–10 months). In one patient which involvement was diffuse and bilateral, the disease relapsed repeatedly with an abscess formation. At first she was reluctant for use of corticosteroid but obliged to receive because her quality of life was very affected. We administrated corticosteroid with a dose of 16 mg prednisone twice a day but could not continue because of glucose intolerance.

## Discussion

IGM, is a rare benign inflamatory breast disease mostly seen in females at a reproductive age [3]. In our study, all but three of patients were at retroductive age and all patients were parous. Many agents, such as local irritants, oral contraseptive pill, viruses, mycotic and parasitic infections, hyperprolactinemia, diabetes mellitus, smoking, alfa 1 antitrypsin deficiency, otoimmunity have been proposed to explain the etiology of IGM but never proven [3-8].

IGM presents most commonly with a painful, firm, tender, ill defined mass in the breast and unilateral [1,8]. The lesions may be located in any quadran of the breast [8]. In consequence of granulomatous inflammation, IGM can cause skin thickness, sinus and abscess formation, axillary lymphadenopathy and nipple retraction which may be clinically mistaken for breast carcinoma [1,3,8]. All patients were admitted with painful mass to our institution, 66.6% of these accompanied with skin changes. The lesions were located in any location but there were the tendency the subareolar and diffuse involvement with percentage of 41.7%. Thus bilateral involvement is reported very rare [6,8,9], there were two patients in our series with bilateral and diffuse involvement. All masses were firm and ill defined and enlarged axillary lymph nodes were present in 14 (41.7%) patients, but all of these enlarged nodes were established reactive and not suspicious for malignancy on US.

US and MMG identified an irregular and ill defined mass in the majority of patients. The information, obtained from US and MMG is non-spesific and lack of specificity to diagnose IGM or to exclude breast carcinoma. Memiş et al. reported the appearance of irregular hypoechoic mass lesions and tubular hypoechoic areas connecting to the mass as frequent sonographic features [10]. In other studies, parenchimal heterogeneity and areas of mixed echo pattern have been reported [7]. Distinct from these lesions enlarged axillary lymph nodes give rise to thought a locally advanced breast carcinoma [7]. In our study, the most common US findings were parenchimal heterogeneity, irregular hypoechoic mass and abscess formation. 41.7% of patients presented enlarged axillary lymph nodes, but these nodes did not show malignant criteria such as cortical thickness (cut-off 2.5 mm) and the absence of the fatty hilum [11] on US. To our opinion, while physical examination give rise to thought a locally advanced breast carcinoma, the appearance of parenchimal heterogeneity and abscess formation on US especially with enlarged reactive axillary lymph nodes support the presence of an inflammatory granulomatous process. But these findings are non-specific and do not exclude carcinoma. Hereby, histologic confirmation is mandatory to ensure that malignancy is not missed.

An ill defined mass, asimmetrically increased diffuse or focal density without parenchimal distortion or microcalcification are the most common findings on MMG [7,12]. Concordant with previous report asymmetric density was detected in our all patients.

According to some studies, MRI does not provide adjunct information for the differentiation of IGM from carcinoma [7]. But Dursun et al. reported that the kinetic features are usually nonsuspicious and can be helpful in the differential diagnosis from carcinoma [12]. But MRI does not play a role in the differential diagnosis between other inflammatory and granulomatous diseases and IGM [1,8,12].

These above mentioned imaging modalities are non-spesific enough, faithful to diagnose IGM and cannot differentiate IGM from carcinoma and other causes of granulomatous and inflammatory lesions such as bacterial mastitis, tuberculous mastitis, foreign body granulomas, sarcoidosis and Wegener ‘s granulomatosis [8]. Since pyhsical examination and imaging modalities fail to diagnose and differential diagnose, histopathologic diagnosis must be performed. Histopathologic diagnosis can be achieved with fine needle aspiration cytology (FNAC), core, incisional or excisional biopsy [7]. But the usefulness and reliability of FNAC has been debated, because some authors informed the useful role of FNAC and the others expressed that the various causes of granulomatous inflammation cannot be differentiated with FNAC [6,13,14]. In our patients, the histopathologic diagnosis was obtained from excisional and incisional biopsies, we have not prefferred FNAC.

The optimal treatment of IGM remains controversial. Surgical excision stil seems to be the best treatment. Wide local excision can be appropriate treatment also provide exact diagnosis and treatment. After wide local excision, if possible, further therapy is not needed. Different recurrence rates (range 5.5%-50%) are reported after wide local excision [1,8]. There was one (6.6%) recurrence of 15 patients that wide local exsicion was performed in our patients. Our recurrence rate is lower than reported rates [11].

According to our opinion, there is no problem if wide local excision can be performed. As a matter of fact, complicated IGM with abscess, fistula or diffuse involvement poses the problem. There is not ideal treatment for complicated IGM. In these patients, wide local excision cannot be achieved. Lai et al.reported that spontaneous resolution occured in 50% of cases of IGM without any treatment with a mean interval of 14.5 months [15]. Also, we had 9 patients which wide local exision could not be performed because of abscess formation and/or diffuse involvement. The lesions of the patients all but one resolved with a mean interval of 5.1 months (range 3–10 months). In one patient which involvement was diffuse and bilateral the disease relapsed repeatedly with an abscess formation. There was not recurrence in the other patients with abscess formation.

Some authors recommended to reduce the size for complicated and resistant cases or diffuse lesions before excision [1]. But there has been reluctance for side effects. We suggested steroid to our complicated patients after abscess drainage and antibiotics, but any patient accepted by reason of side effects, they preffered conservative treatment with close follow-up.

We consider that wide local excision is the better treatment of choice of IGM if possible. We hesitate to recommend corticosteroids in complicated cases for several reasons. First, there is no consensus about timing, duration and dose of corticosteroid administration. Second, according to the study of Sakurai et al., complete resolution of mass lesions with corticosteroid was achieved from 4 to 10 months [2]. Moreover spontaneous resolution occured with a mean interval of 3.3 months in the patients with abscess drainage in our study. But clinical presentation of patient population was different in above mentioned study and in our series. Accordingly it is necessary to know if corticosteroid is proven to rapidly resolve lesions than conservative approach. According to us it is important to keep in mind that spontaneous resolution may occur rapidly contrary to expectations. At least, we consider that corticosterod administration may be suspended after a short follow-up period to see if spontaneous resolution is present or not.

There is still not any commonly accepted optimal treatment for IGM. Wide local excision and corticosteroid administration has been reported for the first line treatment of IGM. The wide spectrum of clinical signs, symptoms and course of IGM make it difficult to choose and standardise a favorable treatment. Which one of the treatment modalities should be favorable? When should wide local excision or corticosteroid be preferred? Is it true to administrate the same dose or duration of corticosteroids for any patient? What should be done if patient is reluctant to corticosteroid?

It is clear there is a need of prospective randomized trials comparing conservative approach, wide local excision and corticosteroid administration to optimize and standardize the diagnosis and treatment of IGM. Nevertheless, it may be possible to compose an algorithm which is mandatory for treatment of IGM.

## Conclusion

IGM is a rare benign inflammatory breast disease that may be misdiagnosed as breat carcinoma. Because of clinical and imaging diagnosis has often been difficult, the definitive diagnose must be obtained from histopathological diagnose.

There is still no commonly accepted treatment for IGM. We consider that wide local excision is the better treatment choice if possible. But, complicated IGM with abcess, fistula and diffuse involvement poses the problem. In this study we observed that spontaneous resolution may occur in these patients and recurrence is not higher from other series.

## Competing interests

The authors declare that they have no competing interests.

## Authors’ contributions

GK and EGD have made substantial contributions to conception and design and written the manuscript. SA, MT and AG have made analysis and interpretation of data. IK and ABÖ have been involved in drafting the manuscript or revising it critically for important intellectual content. MK have given final approval of the version to be published. All authors read and approved the final manuscript.

## Pre-publication history

The pre-publication history for this paper can be accessed here:

http://www.biomedcentral.com/1471-2482/14/66/prepub
